# Serial exercise testing in children, adolescents and young adults with Senning repair for transposition of the great arteries

**DOI:** 10.1186/1471-2261-12-88

**Published:** 2012-10-15

**Authors:** Roselien Buys, Werner Budts, Tony Reybrouck, Marc Gewillig, Luc Vanhees

**Affiliations:** 1 Department of Rehabilitation Sciences, Research Center for Cardiovascular and Respiratory Rehabilitation, KULeuven, Tervuursevest 101, Leuven, 3001, Belgium; 2 Department of Cardiology, University Hospital Gasthuisberg, Herestraat 49, Leuven, 3000, Belgium; 3 Department of Cardiovascular Rehabilitation, University Hospital Gasthuisberg, Herestraat 49, Leuven, 3000, Belgium; 4 Department of Pediatric Cardiology, University Hospital Gasthuisberg, Herestraat 49, Leuven, 3000, Belgium

**Keywords:** Exercise capacity, Transposition of the great arteries, Senning repair, Median term follow-up

## Abstract

**Background:**

Patients with Senning repair for complete transposition of the great arteries (d-TGA) show an impaired exercise tolerance. Our aim was to investigate changes in exercise capacity in children, adolescents and adults with Senning operation.

**Methods:**

Peak oxygen uptake (peak VO_2_), oxygen pulse and heart rate were assessed by cardiopulmonary exercise tests (CPET) and compared to normal values. Rates of change were calculated by linear regression analysis. Right ventricular (RV) function was assessed by echocardiography.

**Results:**

Thirty-four patients (22 male) performed 3.5 (range 3–6) CPET with an interval of ≥ 6 months. Mean age at first assessment was 16.4 ± 4.27 years. Follow-up period averaged 6.8 ± 2 years. Exercise capacity was reduced (p<0.0005) and the decline of peak VO_2_ (−1.3 ± 3.7 %/year; p=0.015) and peak oxygen pulse (−1.4 ± 3.0 %/year; p=0.011) was larger than normal, especially before adulthood and in female patients (p<0.01). During adulthood, RV contractility changes were significantly correlated with the decline of peak oxygen pulse (r= −0.504; p=0.047).

**Conclusions:**

In patients with Senning operation for d-TGA, peak VO_2_ and peak oxygen pulse decrease faster with age compared to healthy controls. This decline is most obvious during childhood and adolescence, and suggests the inability to increase stroke volume to the same extent as healthy peers during growth. Peak VO_2_ and peak oxygen pulse remain relatively stable during early adulthood. However, when RV contractility decreases, a faster decline in peak oxygen pulse is observed.

## Background

Until about 1980, most children with complete transposition of the great arteries (d-TGA) underwent the Mustard or the Senning operation. These atrial switch techniques were replaced when an arterial switch procedure was introduced which allows for a systemic left ventricular function. However, 75% to 80% of the patients with Mustard or Senning repair have survived into adulthood and are now regularly visiting the outpatients clinics for grown-up congenital heart disease [1,2].

It has often been shown that children, adolescents and adults with Senning repair for d-TGA have a substantially reduced exercise capacity, which probably lowers faster with age compared to healthy controls [2-8]. This lower exercise capacity has been explained by chronotropic incompetence, impaired systemic ventricular function, impaired function of the intra-atrial conduit, the inability to increase stroke volume, impaired lung function with ventilation/perfusion mismatch and peripheral deconditioning [2,6,7]. Moreover, a poor exercise capacity identifies d-TGA patients at risk for hospitalization and death [9].

Cross-sectional studies show evidence for a general decrease in exercise capacity in patients with atrial switch repair for d-TGA [4,6]. Reybrouck et al. performed a longitudinal study in children and adolescents with atrial switch operation [3]. They concluded that in the overall group, the exercise performance remained stable during a follow-up of 3.5 ± 2 years, but that in individual patients, a decreasing exercise capacity was correlated with the development of hemodynamic lesions [3].

At present, information on the evolution of the exercise capacity in adult patients with Senning operation performed during childhood is not available and possible differences in the evolution of the exercise capacity between children and adults have not been investigated yet.

Therefore the aim of our study was to investigate the change of the exercise capacity and the cardiovascular status in children, adolescents and young adults with Senning repair for transposition of the great arteries.

## Methods

### Patients

All patients with Senning repair for d-TGA who performed at least 3 cardiopulmonary exercise tests until exhaustion, at least 6 months apart from each other were included in the serial study. Their exercise tests were performed at the occasion of their follow-up visits to the outpatient clinic of paediatric cardiology or adult congenital heart disease of our hospital, therefore no further written informed consent was needed according to the regulations of the ethic committee. The study was approved by the Institutional Review board of the University Hospitals Leuven.

### Cardiopulmonary exercise tests

Patients younger than 16 years old performed a graded maximal exercise test on the treadmill. The treadmill was set at a speed of 5,6 km/h. The gradient was increased every minute by 2% until exhaustion was reached. The children were encouraged to perform a true maximal effort. Oxygen uptake and carbon dioxide output were measured on a breath-by-breath basis by a computerised system with fast responding electronic gas analysers (Med Graphics, Ultima). Inspiratory and expiratory flow was measured with a Pitot flow meter. The system was calibrated before each exercise test with test gas of known composition. Heart rhythm was continuously monitored during exercise and a twelve-lead electrocardiogram was recorded every minute. Patients' results were compared with reference values and expressed as percentage of normal. The normal values were obtained from a large cohort of healthy school children (n=234, male/female, age range 5.7 - 18.5 years) who performed the same exercise testing protocol [10].

Patients from 16 years and older performed maximal exercise tests on a bicycle ergometer (Ergometrics 800S, Ergometrics, Bitz, Germany). The initial workload of 20W was increased by 20W every minute until exhaustion. Patients were encouraged to perform a true maximal effort. A twelve-lead electrocardiogram and respiratory data through breath-by-breath analysis were continuously registered with a computerized system (Oxygen AlphaR, Jaeger, Mijnhardt, Bunnik, The Netherlands). The gas analyzers and the flow meter were calibrated before each test according to the manufacturer’s instructions. Oxygen uptake (VO_2_) was determined from the continuous measurement of the oxygen concentration in the inspired and expired air. Results were compared to results of a control group of 368 men and 158 women (age range 16–65 years old) who performed the same exercise testing protocol in our laboratory, and expressed as percentage of normal values according to age, gender and weight.

Exercise performance was assessed by determination of the peak oxygen uptake (peak VO_2_), and peak heart rate (HR). Peak oxygen pulse was calculated by dividing peak VO_2_ expressed in ml/min/kg by peak HR, which was then multiplied by ten in order to obtain values greater than 1.

### Echocardiography

Routine transthoracic echocardiography was performed in all patients at all assessments with standard gray-scale, and Doppler imaging examinations. All echocardiographic studies were performed with the patient in a supine position. As it is general practice, qualitative evaluations were used for the evaluation of right ventricular hypertrophy (0= no hypertrophy, 1=hypertrophy), right ventricular dilatation (no, slight, moderate and severe dilatation, scaling from 1 to 4), right ventricular contractility (normal, mild, moderate, and severe dysfunction, scaling from 1 to 4) and tricuspid regurgitation (0-4/4).

### Statistical analysis

SAS statistical software version 9.3 for windows (Sas Institute Inc, Cary, NC, USA) was used for the analysis. Data are reported as means and standard deviation or as numbers for dichotomous variables. Exercise capacity was compared with average (mean percent predicted value=100%) using a one-sample t-test. Mean rate of change was calculated for each patient by linear regression analysis. The one-sample t-test was again used for comparison of the rate of change with average (mean percent predicted change=0%). Student's paired t-test, or paired sample Wilcoxon signed rank sum test when appropriate, was used to compare differences between measures from initial and final assessments. Pearson or Spearman correlation coefficients were calculated to assess possible relationships between rates of change in exercise measures and age at Senning operation or changes in echocardiographic parameters. All statistical tests were 2-sided with a significance level of ≤0.05.

## Results

### Patients

A total group of 34 patients with Senning operation for d-TGA were included in our study. The characteristics of these patients are represented in Table 1. Age at the initial evaluation ranged between 8 and 23 years and at the last assessment the age of the patients ranged between 13 and 29 years. Median time between the first and the last assessment was 6.6 years and ranged between 1.6 and 11 years. All patients were in New York Heart Association class I at all assessments.

**Table 1:** Patient characteristics (n=34)

**Male**	**22 (66)**
Age at repair (months)	4 (0.4 - 14)
Time between first and last assessment (years)	6.8 ± 2.0
Number of assessments per patient	3.5 (3–6)

*Data are expressed as mean* ± *standard deviation or as median (range).*

### Exercise measures

Exercise data from the first and the last assessment are shown in Table 2. For all assessments, percentages of predicted peak VO_2_, peak HR and peak oxygen pulse were significantly lower than the normal values.

**Table 2:** Comparison of initial with final assessment

	**First test**	**Last test**	**p**
Age (years)	16.4 ± 4.27	22.9 ± 4.30	<0.0001
Height (cm)	161 ± 16	171 ± 11	0.0002
Weight (kg)	51.8 ± 16.1	65.8 ± 11.5	<0.0001
Peak oxygen uptake (ml/min/kg)	33.6 ± 6.65	27.4 ± 6.96	<0.0001
Peak oxygen uptake (%)°	73 ± 12**	65 ± 13**	0.002
Peak heart rate (beats/min)	179 ± 18	174 ± 15	0.022
Peak heart rate (%)°	94 ± 10*	94 ± 8**	0.69
Peak oxygen pulse (ml/kg/beat)	19.0 ± 4.39	15.8 ± 4.01	<0.001
Peak oxygen pulse (%)°	79 ±16**	69 ± 15**	0.002
Right ventricular contractility			
Normal	5	5	
Mildly reduced	22	12	
Moderately reduced	6	17	
Severely reduced	1	0	
Right ventricular dilatation			
Normal	0	1	
Mildly dilated	2	1	
Moderately dilated	30	29	
Severely dilated	2	3	
Tricuspid regurgitation			
0,5-1/4	16	8	
2/4	12	13	
3/4	6	12	
4/4	0	1	

Data are expressed as mean ± standard deviation or as numbers of patients.

°Percentage of predicted values according to age and gender based on own control group.

Significantly different from 100%: * p<0.005; ** p<0.0001.

As shown in Table 3, a significant decline from the initial to the final assessment was found for peak VO_2_ and peak oxygen pulse when expressed as percentages of predicted values (p=0.015 and p=0.011 respectively). When our patient group was divided into two groups based on the age at first assessment, this decrease was only significant in patients younger than 16 years old at first assessment (p<0.01). Further subanalysis by gender revealed a significant decline of peak VO_2_ (decline −3.3 ± 2.3 %/year; p=0.026) and peak oxygen pulse (decline −3.0 ± 3.9 %/year; p=0.022) in female patients but not in male patients.

**Table 3:** Changes in exercise measures in % per year by demographic variables

	**Peak oxygen uptake**	**Peak heart rate**	**Peak oxygen pulse**
All (n=34)	−1.42 ± 3.25*	−0.19 ± 1.22	−1.39 ± 3.0*
Gender			
Male (n=22)	−0.38 ± 1.70	0.15 ± 0.70	−0.50 ± 1.94
Female (n=12)	−3.34 ± 2.35	−0.83 ± 1.69	−3.02 ± 3.91*
Age at first assessment			
<16 years (n=18)	−2.02 ± 4.05*	−0.30 ± 1.20	−1.92 ± 3.64*
≥16 years (n=16)	−0.75 ± 1.95	−0.08 ± 1.27	−0.79 ± 2.02

*Data are expressed as mean* ± *standard deviation.*

* Significantly different from 0% (p<0.05).

**Figure 1:**
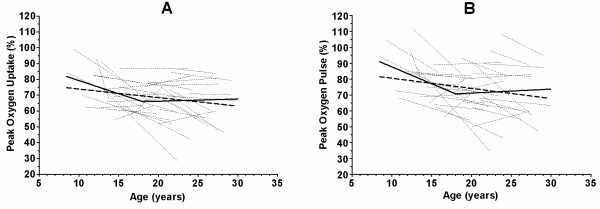
**Change of percentage of predicted peak oxygen consumption (A) and peak oxygen pulse (B) as a function of age.** The thicker line depicts the mean change in percentage of predicted values as calculated by segmental regression analysis in which slope and intercept are calculated before and after the age of 18 years. The thicker dotted line shows the mean percentage of predicted values in the population according to age calculated by linear regression analysis. Thin dotted lines show patient-specific linear regression lines.

Individual changes in peak VO_2_ and peak oxygen pulse expressed as a percentage of the predicted normal value are presented in figure 1. When a cut off point was set at 18 year, the change in peak VO_2_ % and peak oxygen pulse was higher before (p<0.01) then after the cut off point.

Chronotropic incompetence, as defined as a peak HR lower than 80% of the normal value, was only present in 2 patients (6%) at the initial assessment and in 3 patients (9%) at the final assessment. The rate of change in peak HR was not significantly different from a normal population. Rates of change in peak VO_2_, peak HR and peak oxygen pulse were not related to age at surgical repair.

### Echocardiography

Echocardiographic findings are summarized in Table 2. At the first assessment all patients had RV hypertrophy, most patients showed mildly reduced RV contractility and moderate RV dilation and all patients had some degree of tricuspid regurgitation. During the follow-up period, a decrease in RV contractility was reported in 10 patients, one patient showed an increase in RV dilatation, and 7 patients developed a worsening of the tricuspid valve incompetence.

During adulthood, the difference in RV contractility between the last and the first assessment was significantly correlated with the change in peak oxygen pulse (r= −0.5; p=0.047).

## Discussion

This study shows at first that patients with Senning operation for d-TGA have a lower exercise capacity, as expressed by peak VO_2_, peak oxygen pulse and peak heart rate, than what would be expected in a healthy population. Secondly, peak VO_2_ and peak oxygen pulse decrease faster with age than in healthy controls. This decline is most obvious during childhood and adolescence and in female patients. During early adulthood, peak VO_2_ and peak oxygen pulse show a similar evolution as in healthy peers. Thirdly, a decline in peak oxygen pulse in adults is associated with a decrease in RV contractility.

In previous research, several investigators have observed a lower exercise capacity in patients with atrial switch operation for d-TGA compared to a normal population and our results are in agreement with these observations [2,6-8,11]. The general decline of peak VO_2_ with age is well known [3,6,12]. Budts et al. suggested that this decrease in peak VO_2_ might be faster in adults with atrial switch repair since a strong negative relationship was found between cardiac index during submaximal exercise and age [7]. Our results showed a significantly faster decline of peak VO_2_ in our patients compared to the normal evolution, and our study thus provides some evidence for this reasoning. A possible explanation for a faster decline in exercise tolerance is the fact that these patients have a systemic RV, which is not designed to support the systemic circulation. The incapacity of the RV to increase stroke volume at higher exercise intensities was already shown to be probably causing reduced exercise capacity in patients with Senning repair [7]. An impaired atrio-ventricular transport which leads to a poor filling of the ventricle, and poor quality of the right ventricle and the valves might be one of the main determinants of an impaired stroke volume response to exercise [7]. It seems that the RV cannot keep performing against systemic pressures in the same manner as the left ventricle, causing the right ventricular function to decline with age, which at its turn contributes to a decline in exercise capacity. Our results show a weak but significant relationship between a decrease in oxygen pulse and a decrease in right ventricular contractility, supporting this reasoning. During growth, stroke volume increases when body mass increases for the same absolute rate of work. When stroke volume is estimated from the response of the oxygen pulse, our data suggest that children and adolescents with d-TGA cannot increase their stroke volume to the same extent as healthy peers.

Our results suggest that the decrease in peak VO_2_ and peak oxygen pulse occurred during childhood and adolescence, and was more stable during early adulthood. However, we believe that the small number of patients in the older age group resulted in a lack of power and therefore did not result in similar findings as Budts et al. [7]. Since the study population in the earlier mentioned investigation consisted of patients with Senning and Mustard operation, their different findings might also be triggered by possible differences between patients with Mustard and Senning operation. Reybrouck et al. also serially investigated the exercise tolerance, as assessed by the ventilatory threshold and the rate of increase in oxygen uptake, during medium term follow up (about 3 years in average) in children and adolescents with Mustard and Senning repair for d-TGA [3]. They reported that no significant change could be observed for exercise capacity. This is also in contradiction with our results, but we believe that the shorter follow-up period, smaller patient number, different population composition, younger age, other outcome measures and the fact that patients were only tested twice might account for these different findings.

The decrease in aerobic capacity in children and adolescents with atrial switch repair for d-TGA was cross sectionally studied by Fredriksen and colleagues and they also reported a significant decline in aerobic capacity with increasing age in young atrial switch patients [6]. They argued that chronotropic incompetence may be one of the reasons for this diminishing exercise capacity [6]. However, in our patient cohort, peak heart rate did not decline significantly and chronotropic incompetence was only present in a few patients. It is therefore unlikely that this might have influenced the overall change of the exercise capacity in our study group.

Furthermore, in patients with Senning operation, a reduced exercise capacity is often contributed to a lack of physical activity, which might be based on overprotection by the environment during childhood [13]. If patients with d-TGA have a low physical activity level during childhood and adolescence, this might counteract with the normal development of their exercise capacity and therefore explain the inability to increase the exercise capacity during growth to the same extent as healthy peers [13]. Moreover, it has recently been shown that adults with atrial switch repair for d-TGA show a reduced physical activity level in comparison with healthy counterparts, which is related to a lower exercise capacity [14]. Even though our data do not show a faster decline of the exercise capacity in young adults, we believe that on the longer term this sedentary lifestyle will probably contribute to the evolution of the exercise intolerance, making it decline faster than in a healthy and generally more active population.

Nowadays, the Senning operation is abandoned for routine palliation and replaced by the arterial switch operation [15]. However, Senning patients are still visiting specialized centres for adult congenital heart disease and often present with decreased systemic ventricular function and/or systemic atrioventricular valve regurgitation, which might deteriorate further with age and can lead to a larger decline of peak oxygen uptake and peak oxygen pulse than in a normal population. The care of patients with Senning operation for d-TGA who are growing older will therefore remain a huge challenge for the specialized cardiologists of our era in order to ensure the patients' health, well-being and quality of life.

### Study limitations

A first limitation of our study is the low number of patients which does not allow that general conclusions are drawn. Secondly, our results can not be applied to patients older than 30 years of age. Thirdly, echocardiography has his limitations for the assessment of right ventricular function and size and our results remain to be confirmed by evaluations based on stronger cardiac imaging methods [16]. And finally, different exercise modalities were used for the children and the older patients. We are aware of the fact that results might be influenced by these differences; however we believe we take that into account by only working with percentages of predicted values in our analyses.

## Conclusions

Patients with Senning operation for d-TGA have a lower exercise tolerance compared to a healthy population. Peak VO_2_ and peak oxygen pulse decline faster with age compared to healthy controls. This progressive decline is most obvious during childhood and adolescence and suggests the inability to increase stroke volume to the same extent as healthy peers during growth. Peak VO_2_ and peak oxygen pulse remain relatively stable during early adulthood. However when RV contractility decreases, a faster decline in peak oxygen pulse is observed.

## Abbreviations

d-TGA: Complete transposition of the great arteries; peak VO_2_: Peak oxygen uptake; CPET: Cardiopulmonary exercise tests; RV: Right ventricle/ventricular; HR: Heart rate.

## Competing interests

The authors declare that they have no competing interests.

## Authors' contributions

BR collected, cleaned and analyzed data, and she drafted and revised the manuscript. RT collected data and revised the manuscript. BW, GM and VL participated in the design of the study and revised the draft paper. All authors read and approved the final manuscript.

## Pre-publication history

The pre-publication history for this paper can be accessed here:

http://www.biomedcentral.com/1471-2261/12/88/prepub
